# Rasch Validation of the FRTVMI in Arab Adolescents: Cultural and Gender Considerations in Visual–Motor Assessment

**DOI:** 10.1155/oti/8417993

**Published:** 2026-04-19

**Authors:** Ehab Mohamed Naguib Omara, Amthal H. Al-Huwailah, Mahmoud Mohamed Emam, Atef Mosaad Elsherbiny, Asma A. Al-Attiyah, Ali Mahdi Kazem, Al-Ghaliya Harith Sulaiman Al Hinai

**Affiliations:** ^1^ Department of Psychology, College of Education, Sultan Qaboos University, Seeb, Oman, squ.edu.om; ^2^ College of Social Sciences, Kuwait University, Kuwait City, Kuwait, kuniv.edu; ^3^ Department of Psychological Sciences, College of Education, Qatar University, Doha, Qatar, qu.edu.qa

**Keywords:** construct validity, Full Range Test of Visual–Motor Integration, Gulf countries, Rasch model

## Abstract

This study examined the construct validity of the Full Range Test of Visual–Motor Integration (FRTVMI) among adolescents aged 11–16 years in Oman, Qatar, and Kuwait using the Rasch measurement model. A total of 2464 students were selected through cluster random sampling to ensure national representation. Rasch analysis evaluated the unidimensionality, item fit, local independence, scalability, and reliability of the test. Results indicated strong evidence of unidimensionality, high item reliability, and satisfactory person reliability across samples. However, notable discrepancies in item difficulty ordering emerged between countries and relative to the original test manual, suggesting cultural and educational influences on item performance. Differential item functioning (DIF) analysis identified several gender‐biased items—most prominently in the Kuwaiti sample—raising concerns about test fairness. While the FRTVMI demonstrates strong psychometric properties, the findings highlight the need for culturally responsive adaptations and further research to enhance its validity and equity across diverse populations in the Gulf region.

## 1. Introduction

Visual–motor integration (VMI) is a fundamental neurocognitive process involving the coordinated interaction between visual perception and motor control, enabling individuals to translate visual input into precise and goal‐directed motor actions. This coordination is central to a wide range of academic and everyday activities, including drawing, handwriting, object manipulation, and the construction of geometric forms [1]. VMI develops through the dynamic integration of sensory perception, fine motor coordination, and visual processing, forming a core spatial cognitive ability that bridges perceptual, motor, and spatial reasoning domains [2, 3]. Its importance is reflected in daily functional tasks such as handwriting [4], using utensils, tying shoelaces, performing finger‐based arithmetic, and constructing geometric figures [5]. Recent research has further emphasized the contribution of visual perceptual functions—such as binocular vision and contrast sensitivity—to fine motor precision, demonstrating that enhanced visual processing can reduce errors and increase efficiency in tasks involving small object manipulation [6]. Collectively, these findings underscore the central role of VMI in supporting both academic achievement and functional independence.

The significance of VMI spans the entire developmental continuum. In early childhood, VMI represents a foundational capacity underlying fine motor coordination, handwriting development, and the emergence of numerical cognition. Empirical evidence suggests that fine motor proficiency, in conjunction with executive functions such as working memory, indirectly contributes to mathematics achievement by facilitating basic skills such as magnitude comparison [7]. During this stage, foundational motor abilities begin to emerge through activities requiring coordinated visual–motor processing, including writing, drawing, reading, and manipulation of small tools [8–10]. As children progress developmentally, the cognitive and motor demands placed on VMI increase. For example, handwriting evolves from the isolated formation of letters to the fluent integration of letters into words and sentences, requiring increasingly refined coordination between visual perception and fine motor execution [1]. Assessing VMI during this period provides critical insights into children′s learning trajectories and supports early identification of those who may benefit from targeted educational or therapeutic interventions.

VMI is also closely linked to developmental disorders that affect motor coordination and learning. Children with autism spectrum disorder (ASD), for instance, frequently exhibit difficulties in manual dexterity and dynamic object manipulation, such as catching or aiming, reflecting broader impairments in motor coordination and VMI [11]. These challenges highlight the importance of reliable and sensitive VMI assessment tools that can inform individualized intervention planning. Beyond childhood and adolescence, VMI continues to play a critical role in functional independence during adulthood. Disruptions in VMI are commonly observed following neurological events such as mild traumatic brain injury (mTBI), where individuals often experience reduced hand–eye coordination and difficulty performing everyday tasks independently. Such deficits have been linked to impaired integration within frontoparietal neural networks responsible for visuospatial processing and motor control [12]. In clinical practice, VMI assessments are widely used to evaluate impairment severity and monitor rehabilitation progress, particularly in occupational and neurocognitive therapy settings [13].

Recent longitudinal research has further highlighted the prognostic relevance of VMI in aging populations. Declines in VMI have been identified as early indicators of mild cognitive impairment (MCI) and Alzheimer′s disease, with evidence linking these declines to diminished visuospatial processing and executive functioning—both of which are integral components of VMI performance [3]. Importantly, intervention studies have demonstrated that multidomain motor–cognitive training programs can lead to measurable improvements in VMI among older adults with MCI, reinforcing its value as both a diagnostic and predictive construct. Such findings emphasize that VMI represents a lifelong functional capacity with relevance for cognitive health, autonomy, and quality of life.

Given its broad developmental and clinical significance, the assessment of VMI has long relied on standardized instruments designed to capture the integration of visual perception and motor execution. These instruments generally fall into two categories. Motor‐free visual perception tests assess visual processing abilities—such as spatial relations and visual discrimination—without requiring direct motor output [14]. In contrast, figure‐copying tasks provide a more direct assessment of VMI by requiring individuals to reproduce geometric forms of increasing complexity. Among the most widely used figure‐copying instruments are the Beery–Buktenica Developmental Test of Visual–Motor Integration (Beery VMI) and the Full Range Test of Visual–Motor Integration (FRTVMI) [15–17]. These measures are extensively applied in educational, clinical, and research settings to evaluate VMI across childhood, adolescence, and adulthood. These distinctions highlight the importance of selecting assessment tools that not only capture visual–motor performance but also provide psychometrically sound and interpretable measurement across diverse populations.

The FRTVMI was developed as an advanced iteration of earlier VMI measures, with the aim of addressing several psychometric limitations of its predecessors. Notably, it extends normative coverage across a wide age range and demonstrates improved measurement precision. Evidence from empirical studies supports the FRTVMI′s strong internal consistency, high interrater reliability, and convergent validity with other established VMI instruments [18, 19]. According to Hopkins′ [20] classification, the reported associations between the FRTVMI and related measures represent large to very large effect sizes, supporting the instrument′s construct validity. Additional studies conducted in Australia and other contexts have further reinforced the FRTVMI′s reliability and clinical utility across diverse populations [18, 21, 22].

Despite its international adoption, the application of the FRTVMI in Arab contexts has remained relatively limited. Existing studies in the Gulf region have provided preliminary evidence supporting its validity among children and adolescents, as well as its association with academic outcomes ([9]; Al‐Attiah and Emam 2020). However, these investigations have largely relied on classical test theory (CTT)–based analyses, which primarily yield global indices such as total scores and internal consistency coefficients. Although such indices are informative, CTT offers limited insight into item‐level functioning, the hierarchical ordering of item difficulty, and the invariance of item parameters across cultural or demographic groups. This limitation becomes particularly important in cross‐national contexts such as the Gulf region, where differences in educational practices, familiarity with geometric representations, or learning experiences may influence how individual test items function. Under CTT, such influences may remain obscured because analyses are typically based on total scores rather than item‐level calibration [23, 24]. As a result, it remains unclear whether the hierarchical ordering of FRTVMI items is stable across Arab contexts or whether certain items function differently across demographic groups such as gender.

Addressing these unresolved questions requires analytic approaches capable of examining item functioning directly. Rasch measurement provides such an approach by enabling the estimation of item difficulty and person ability on a common scale, as well as the evaluation of measurement invariance through differential item functioning (DIF) analysis [23, 25]. These capabilities allow researchers to determine whether observed differences in test performance reflect genuine differences in VMI ability or arise from context‐specific item functioning ([24]; Hambleton and Swaminathan 2010).

To address these methodological limitations, the present study adopts the Rasch measurement model as a complementary analytic framework. The Rasch model, situated within the broader family of item response theory models, provides a probabilistic approach for simultaneously estimating person ability and item difficulty on a common logit scale, thereby enabling the transformation of ordinal raw scores into interval‐level measures (Hambleton and Swaminathan 1985; [23]). Beyond providing reliability indices analogous to those obtained under CTT, Rasch analysis allows for the evaluation of fundamental measurement assumptions, including unidimensionality, local item independence, and the hierarchical ordering of item difficulty [24, 25].

Importantly, Rasch modeling facilitates item‐level calibration and the examination of DIF, which are essential for assessing measurement invariance and fairness across demographic and cultural groups—issues that cannot be adequately addressed using CTT alone [23]. These analytic capabilities are particularly relevant in cross‐cultural assessment contexts, where observed score differences may otherwise reflect measurement artifacts rather than substantive differences in the underlying construct [24].

Accordingly, applying Rasch analysis to the FRTVMI allows the present study to examine whether the hierarchical ordering of items is consistent across Gulf contexts and to evaluate whether any items function differently across gender groups.

By applying the Rasch model to adolescent samples from Oman, Qatar, and Kuwait, the current study is aimed at evaluating whether the FRTVMI functions as a culturally invariant and psychometrically robust measure of VMI. Integrating classical descriptive indicators with Rasch‐based measurement evidence allows for a comprehensive evaluation of the FRTVMI that is both methodologically rigorous and practically interpretable for educational and clinical assessment within diverse Gulf contexts.

## 2. Method

### 2.1. Participants

The study sample consisted of 2464 school‐aged adolescents (49.72% male and 50.28% female) between the ages of 11 and 16 years (M = 13.33, SD = 1.25). Participants were selected through cluster random sampling from public schools across three Arab Gulf countries: Oman (26.38%), Qatar (40.67%), and Kuwait (32.95%). Cluster sampling was chosen due to its practicality and efficiency in educational research settings where students are naturally grouped within schools and classrooms. The final sample included students enrolled in Grades 5–9. Table 1 summarizes key demographic characteristics, including age, gender distribution, grade level, and country of origin.

**Table 1:** Demographic characteristics of participants (*n* = 2464).

Characteristics	
Age mean (standard deviation)	13.33 (1.25)
Gender *N* (%)	
Male	1225 (49.72%)
Female	1239 (50.28%)
Grade *N* (%)	
5	199 (8.08%)
6	456 (18.51%)
7	579 (23.50%)
8	629 (25.53%)
9	601 (24.39%)
Country *N* (%)	
Oman	650 (26.38%)
Qatar	1002 (40.67%)
Kuwait	812 (32.95%)

### 2.2. Data Collection

#### 2.2.1. The FRTVMI

The FRTVMI, 11–74 years version, is a standardized assessment consisting of 18 geometric figures presented in a booklet format. Participants are instructed to copy each figure directly below the corresponding model. The test duration typically ranges from 10 to 30 min, depending on factors such as age, drawing precision, and completion speed. Scoring requires an additional 15 min on average.

All procedures in the current study were conducted in accordance with ethical standards, with approval obtained from the Human Ethical Advisory Boards affiliated with the authors′ institutions. Information regarding the study was formally communicated to local education authorities. In addition to the performance tasks, the test booklet included a brief demographic section to collect participants′ background information.

Participants were recruited through cluster random sampling to ensure a demographically and geographically diverse sample. A total of 10 public schools were selected to represent a range of socioeconomic contexts and regions. Notably, the FRTVMI was introduced to the Gulf region by the third author as part of a nationally funded research project on learning disabilities in the Sultanate of Oman, supported by His Majesty′s Research Trust Fund.

Test administrations took place in regular classroom environments under standardized lighting and testing conditions. The FRTVMI was administered by three trained research assistants, each of whom received country‐specific training from the authors. Prior to data collection, all assistants were required to demonstrate proficiency and fidelity in administering the test according to the prescribed guidelines. Participants were allowed unlimited time to complete the test to accommodate variability in individual pacing.

Upon completion, the FRTVMI data were coded and organized according to participant gender, country, and grade level. Data analysis was conducted using the Statistical Package for the Social Sciences (SPSS), Version 25, and WINSTEPS Version 3.80.1, which was used for Rasch model analysis.

### 2.3. Data Analysis

Data from the FRTVMI were analyzed using WINSTEPS Version 3.80.1, applying the Andrich Rating Scale Model (RSM). This model was employed because all items shared a common four‐category response structure (scores ranging from 0 to 3), which is consistent with the assumptions of the rating scale formulation of the Rasch model. Rasch analysis was used to evaluate item‐level functioning and to examine the construct validity and measurement precision of the FRTVMI.

Following standard Rasch measurement procedures, the analysis was conducted sequentially to evaluate key measurement properties of the scale. Specifically, dimensionality and local item independence were first examined to determine whether the items measured a single underlying construct. Item fit statistics were then evaluated to assess the extent to which individual items conformed to model expectations. Subsequently, person and item reliability and separation indices were calculated to examine the precision of measurement and the scale′s ability to differentiate between levels of VMI ability. Finally, item difficulty hierarchy and DIF were examined to evaluate the stability of item calibration and the presence of potential item bias across demographic groups.

Unidimensionality was evaluated using Principal Component Analysis (PCA) of standardized residuals, which serves as a diagnostic tool for identifying potential secondary dimensions after extraction of the primary Rasch dimension. In this context, the eigenvalue of the first residual contrast was examined as an indicator of unexplained variance. Following Dabaghi et al. [26], eigenvalues below 2.0 were interpreted as indicating an absence of substantial secondary dimensions rather than definitive evidence of strict unidimensionality. Local item independence was evaluated indirectly through inspection of residual correlations, with no substantial local dependencies observed.

Item and person reliability and separation indices were computed to assess the consistency of measurement and the scale′s ability to differentiate among respondents with varying levels of ability and items with differing levels of difficulty. Reliability values were interpreted according to the classification proposed by Leung et al. [27], whereby values below 0.67 were considered low, values between 0.67 and 0.80 sufficient, values between 0.81 and 0.90 good, values between 0.91 and 0.94 very good, and values above 0.94 excellent.

To evaluate item functioning and detect anomalous response patterns, infit and outfit mean‐square statistics were examined. Infit statistics were emphasized, as elevated infit values indicate unexpected response patterns among individuals whose ability levels are well matched to item difficulty and therefore pose a greater threat to construct validity than extreme responses reflected by outfit statistics [25]. Consistent with established guidelines, infit and outfit mean‐square values between 0.6 and 1.4 were considered acceptable [23]. Item difficulty estimates were expressed in logits and visualized using person–item maps to assess the alignment between item difficulty and respondent ability levels.

To examine potential gender‐related item bias, DIF analysis was conducted using both the Rasch–Welch *t*‐test and the Mantel–Haenszel (M–H) chi‐square test as implemented in WINSTEPS. These procedures evaluate uniform DIF, that is, systematic group‐related differences in item difficulty that remain relatively constant across the latent trait continuum. Although both procedures are grounded in the Rasch logit‐linear framework, the M–H test is generally more precise when data are complete and evenly distributed across score levels, whereas the Welch *t*‐test is more robust under conditions of sparse data or unequal group sizes [28]. Items were flagged as exhibiting DIF if either test yielded a significance level of *p* < 0.05, with substantive interpretation guided by the magnitude of DIF according to ETS classification criteria [29]. Specifically, absolute DIF values ≥ 0.64 were classified as moderate to large (Category C), values between 0.43 and 0.63 as slight to moderate (Category B), and values ≤ 0.43 as negligible (Category A). Positive DIF values indicated items favoring the focal group (girls), whereas negative values indicated items favoring the reference group (boys).

## 3. Results

### 3.1. Descriptive Statistics

Descriptive analyses of the raw FRTVMI data were conducted using the SPSS, Version 25. Table 2 presents key descriptive statistics for the scale items across the three national samples—Oman, Qatar, and Kuwait—including means, standard deviations, skewness, and kurtosis. Skewness and kurtosis coefficients were evaluated to examine the distributional properties of item responses. For the Omani sample, skewness ranged from −1.51 to 0.55 and kurtosis from −1.24 to 2.00. In the Qatari sample, skewness ranged from −1.25 to 1.32 and kurtosis from −1.18 to 0.74. For the Kuwaiti sample, skewness values ranged from −1.96 to 1.11 and kurtosis from −0.99 to 1.96. All values were within the commonly accepted range (±2), suggesting approximate univariate normality of item responses across all three samples.

**Table 2:** Descriptive statistics for FRVMI′s scale items across the three countries.

Name	Mean			Std. dev.			Skewness			Kurtosis		
	OM	QA	KW	OM	QA	KW	OM	QA	KW	OM	QA	KW
FRT1	2.38	2.50	2.16	0.64	0.71	0.79	−0.66	−1.25	−0.46	−0.06	0.74	−0.77
FRT2	2.51	2.30	2.18	0.61	0.78	0.79	−0.98	−0.85	−0.51	0.56	−0.03	−0.70
FRT3	2.49	2.10	2.09	0.75	1.05	0.95	−1.49	−0.79	−0.58	1.79	−0.69	−0.87
FRT4	2.40	1.93	2.05	0.70	0.88	0.88	−0.89	−0.15	−0.58	0.19	−1.12	−0.49
FRT5	2.35	1.79	2.58	0.71	0.76	0.80	−0.95	−0.06	−1.96	0.81	−0.52	1.96
FRT6	2.48	2.19	2.09	0.67	0.79	0.87	−1.15	−0.65	−0.61	0.97	−0.25	−0.49
FRT7	2.58	2.41	1.66	0.65	0.80	0.77	−1.51	−1.17	0.17	2.00	0.47	−0.61
FRT8	2.30	1.90	1.61	0.74	0.91	0.84	−0.87	−0.38	−0.08	0.42	−0.74	−0.58
FRT9	2.28	1.65	1.65	0.81	1.03	0.89	−1.04	−0.06	0.21	0.64	−1.18	−0.98
FRT10	2.51	1.47	1.56	0.70	0.91	0.96	−1.51	0.03	0.14	1.96	−0.81	−0.99
FRT11	2.38	1.62	1.47	0.76	0.93	0.84	−1.19	−0.09	0.14	1.15	−0.87	−0.56
FRT12	2.33	1.90	1.82	0.83	0.90	0.87	−1.15	−0.36	−0.11	0.71	−0.76	−0.92
FRT13	2.20	1.59	1.35	0.86	0.96	0.84	−0.87	−0.02	0.41	−0.02	−0.98	−0.38
FRT14	1.96	1.80	1.33	0.86	1.03	0.87	−0.35	−0.28	0.28	−0.74	−1.14	−0.57
FRT15	2.31	1.85	1.58	0.75	0.96	0.89	−0.81	−0.26	0.08	−0.07	−1.02	−0.80
FRT16	1.74	1.64	1.16	1.02	1.01	0.80	−0.44	−0.16	0.38	−0.89	−1.06	−0.24
FRT17	1.88	1.21	0.98	1.10	1.10	0.82	−0.55	0.40	0.61	−1.04	−1.17	−0.03
FRT18	1.04	0.67	0.72	1.15	1.04	0.88	0.55	1.32	1.11	−1.24	0.31	0.41
*Min*	1.04	0.67	0.72	0.61	0.71	0.77	−1.51	−1.25	−1.96	−1.24	−1.18	−0.99
*Max*	2.58	2.50	2.58	1.15	1.10	0.96	0.55	1.32	1.11	2.00	0.74	1.96
*Range*	1.54	1.83	1.86	0.54	0.39	0.19	2.06	2.57	3.06	3.24	1.92	2.95
*FRTVMI* ∗	2.23	1.81	1.67	0.48	0.53	0.51	−0.82	−0.03	0.09	0.19	−0.47	−0.37

*Note:* Asterisk “∗” denotes the descriptive statistics for the raw score of FRTVMI.

The mean item scores also showed variability across countries, ranging from 1.04 to 2.58 in Oman, 0.67 to 2.50 in Qatar, and 0.72 to 2.58 in Kuwait. Importantly, the mean total score for the Omani sample (M = 2.23) was higher than that for Qatar (M = 1.81) and Kuwait (M = 1.67), indicating a comparatively stronger performance on the FRTVMI in the Omani cohort. Standard deviation values were similar across the three samples, reflecting comparable levels of variability in item responses. These distributional findings indicate the absence of extreme skewness or kurtosis and support the stability of item response patterns prior to Rasch analysis.

These descriptive findings indicate that the distributional characteristics of the FRTVMI items were appropriate for subsequent Rasch analysis. Accordingly, the next step of the analysis focused on examining whether the data met the fundamental measurement assumptions of the Rasch model, particularly unidimensionality and local item independence.

### 3.2. Unidimensionality and Local Independence of FRTVMI Items

A principal component analysis of standardized residuals (PCASR) was conducted using WINSTEPS to evaluate the dimensional structure of the 18‐item FRTVMI scale. Within the Rasch measurement framework, examining dimensionality represents an essential step to determine whether the set of items collectively measures a single underlying construct—in this case, VMI. This analysis is aimed at determining whether the scale items collectively measure a single underlying construct—VMI—and assessing the local independence of item responses. As shown in Table 3, the FRTVMI explained a substantial proportion of the total variance in each national sample: 48.5% in Oman, 45.6% in Qatar, and 51.3% in Kuwait. These values suggest that a large share of the variability in item responses is attributable to a dominant latent trait, providing support for the unidimensionality of the scale.

**Table 3:** Principal component analysis of the standardized residuals, reliability, and separation index for persons and items in each country.

Country	Variance explained by measure (%)	Unexplained variance			Reliability		Separation index	
		Total	First cont.	Second cont.	Persons	Items	Persons	Items
Oman	17.0 (48.5%)	18 (51.5%)	1.6 (4.4%)	1.4 (4.1%)	0.85	0.99	2.34	11.62
Qatar	15.1 (45.6%)	18 (54.4%)	1.8 (5.5%)	1.4 (4.3%)	0.86	0.99	2.47	16.99
Kuwait	19.0 (51.3%)	18 (48.7%)	2.1 (5.8%)	1.6 (4.3%)	0.88	0.99	2.77	18.04

The eigenvalues for the first residual contrast—1.6 in Oman, 1.8 in Qatar, and 2.1 in Kuwait—further support this conclusion. In Rasch analysis, the first residual contrast represents unexplained variance after the primary measurement dimension has been extracted. Therefore, examining its magnitude helps identify the potential presence of secondary dimensions. According to Rasch modeling standards, eigenvalues below 2.0 typically indicate acceptable unidimensionality [25]. Although the Kuwaiti sample′s eigenvalue of 2.1 approaches this threshold, it only slightly exceeds the recommended guideline and is therefore interpreted as borderline rather than indicative of a meaningful secondary dimension.

To assess local independence, standardized residual correlations were examined. The maximum correlation observed among residuals in all three samples was below 0.20, which suggests minimal association among item residuals. Low residual correlations indicate that once the primary Rasch dimension has been accounted for, responses to individual items are largely independent from one another. This result indicates that the FRTVMI items function independently, with responses to one item not unduly influenced by responses to another—an essential assumption in Rasch modeling [23].

Taken together, the PCASR results and the residual correlation analysis provide evidence that the FRTVMI operates as a largely unidimensional measure of VMI across the three national samples while also satisfying the Rasch assumption of local item independence.

### 3.3. Item Fit to the Rasch Model

The fit of FRTVMI items to the Rasch model was evaluated by examining infit and outfit mean‐square statistics. Item fit statistics are used in Rasch analysis to determine whether individual items behave in a manner consistent with model expectations, thereby contributing meaningfully to the measurement of the intended construct. According to Linacre [25], high infit mean‐square values are particularly concerning because they may indicate unexpected response patterns among individuals whose ability levels are well matched to item difficulty. Such misfit may suggest that an item does not function consistently across respondents and therefore may threaten measurement validity. Items are generally considered to demonstrate acceptable fit when both infit and outfit mean‐square values fall within the range of 0.6–1.4 ([23]; Boone et al. 2014). Within this range, item responses are regarded as sufficiently predictable according to Rasch model expectations while still allowing for natural variability in observed responses.

As presented in Table 4, the infit mean‐square values ranged from 0.70 to 1.36 in Oman, 0.77 to 1.45 in Qatar, and 0.74 to 1.61 in Kuwait. Similarly, outfit mean‐square values ranged from 0.72 to 1.25 in Oman, 0.83 to 1.44 in Qatar, and 0.73 to 1.47 in Kuwait. Overall, most items demonstrated acceptable fit to the Rasch model across the three national samples, indicating that the majority of items functioned as expected in measuring VMI.

**Table 4:** Rasch item measures and item‐fitting indices across the three countries.

Items	Item difficulty			Infit MSQ			Outfit MSQ		
	OM	QA	KW	OM	QA	KW	OM	QA	KW
FRT1	−0.29	−1.34	−1.01	0.82	1.00	0.99	0.97	0.92	1.09
FRT2	−0.67	−0.87	−1.05	0.92	0.85	0.90	0.99	0.83	0.92
FRT3	−0.61	−0.48	−0.86	1.21	1.45	1.29	1.25	1.44	1.23
FRT4	−0.33	−0.18	−0.78	0.88	0.91	1.09	0.97	1.03	1.11
FRT5	−0.19	0.05	−2.13	0.96	0.77	1.61	1.11	0.98	1.47
FRT6	−0.57	−0.65	−0.86	0.90	0.82	1.03	0.99	0.87	1.14
FRT7	−0.89	−1.1	0.02	0.98	1.05	0.85	0.97	1.00	0.89
FRT8	−0.09	−0.12	0.12	0.70	0.87	0.76	0.72	0.91	0.77
FRT9	−0.04	0.27	0.04	0.90	1.15	0.95	0.96	1.19	0.95
FRT10	−0.67	0.56	0.22	1.01	0.95	1.13	1.00	1.00	1.10
FRT11	−0.29	0.33	0.41	0.92	0.80	0.74	0.92	0.84	0.73
FRT12	−0.15	−0.13	−0.29	1.05	0.92	0.94	0.99	0.92	0.92
FRT13	0.15	0.37	0.64	0.87	0.85	0.86	0.83	0.87	0.85
FRT14	0.64	0.03	0.69	0.82	1.09	0.99	0.88	1.12	0.98
FRT15	−0.11	−0.05	0.19	0.80	0.90	0.89	0.87	0.93	0.91
FRT16	1.05	0.29	1.07	1.15	1.20	0.92	1.20	1.21	0.92
FRT17	0.79	0.99	1.46	1.27	1.18	0.91	1.19	1.13	0.89
FRT18	2.27	2.03	2.13	1.36	1.40	1.40	1.24	1.16	1.32

Only minor deviations were observed. For example, Item FRT3 in the Qatari sample and Item FRT5 in the Kuwaiti sample exhibited slightly elevated fit statistics. However, these deviations were small and did not indicate substantial misfit requiring item removal or modification. Overall, the results suggest that the FRTVMI items largely conform to the expectations of the Rasch measurement model across the three national contexts.

### 3.4. Person and Item Reliability and Separation

Assessment of item and person reliability using the Rasch model revealed high measurement consistency across all three countries. In Rasch analysis, reliability indices reflect the reproducibility of item difficulty estimates and person ability measures if the same sample or items were administered again under comparable conditions. Item reliability was uniformly excellent, with a value of 0.99 for Oman, Qatar, and Kuwait, reflecting strong internal consistency in the performance of test items [23]. Such high item reliability indicates that the sample size was sufficiently large to confirm a stable hierarchy of item difficulty within the scale. Person reliability ranged from 0.85 to 0.88, indicating satisfactory reproducibility of participant ability estimates across the samples [30]. These values suggest that the FRTVMI was able to consistently differentiate participants according to their levels of VMI ability. As shown in Table 3, the item separation index—which reflects the scale′s capacity to distinguish between varying item difficulties—ranged from 11.62 in Oman to 18.04 in Kuwait, indicating excellent differentiation. High item separation values indicate that the items are distributed across multiple levels of difficulty, allowing the scale to establish a stable item hierarchy along the latent trait continuum. The person separation index, which reflects the scale′s ability to discriminate among individuals′ abilities, ranged from 2.34 to 2.77, also indicating strong performance [31]. Person separation values above 2.0 suggest that the instrument can distinguish between at least three statistically distinct levels of respondent ability.

Taken together, the reliability and separation indices indicate that the FRTVMI provides stable item calibration and adequate discrimination among individuals with different levels of VMI across the three national samples.

### 3.5. Person and Item Calibration

Item difficulty parameters for the FRTVMI, presented in logits in Table 4, varied across countries: from −0.89 to 2.27 in Oman, −1.34 to 2.03 in Qatar, and −2.13 to 2.13 in Kuwait. In Rasch measurement, item difficulty estimates expressed in logits represent the relative position of each item along the latent trait continuum, allowing items and persons to be placed on the same measurement scale. Notably, Item 18 was consistently the most difficult across all samples, whereas the least difficult items varied by country: Item 7 in Oman, Item 1 in Qatar, and Item 5 in Kuwait. This variation indicates that although the upper end of the difficulty hierarchy remained stable, differences were observed in the ordering of easier items across national samples. The overall hierarchical ordering of item difficulty was not consistent across the three samples and differed from the sequence outlined in the FRTVMI examiner′s manual [17]. Such discrepancies in item ordering may suggest that contextual or educational factors influence how specific visual–motor tasks are performed across cultural settings. This inconsistency suggests potential cultural or contextual influences on item performance and points to possible redundancy among items of similar difficulty levels.

**Figure 1:**
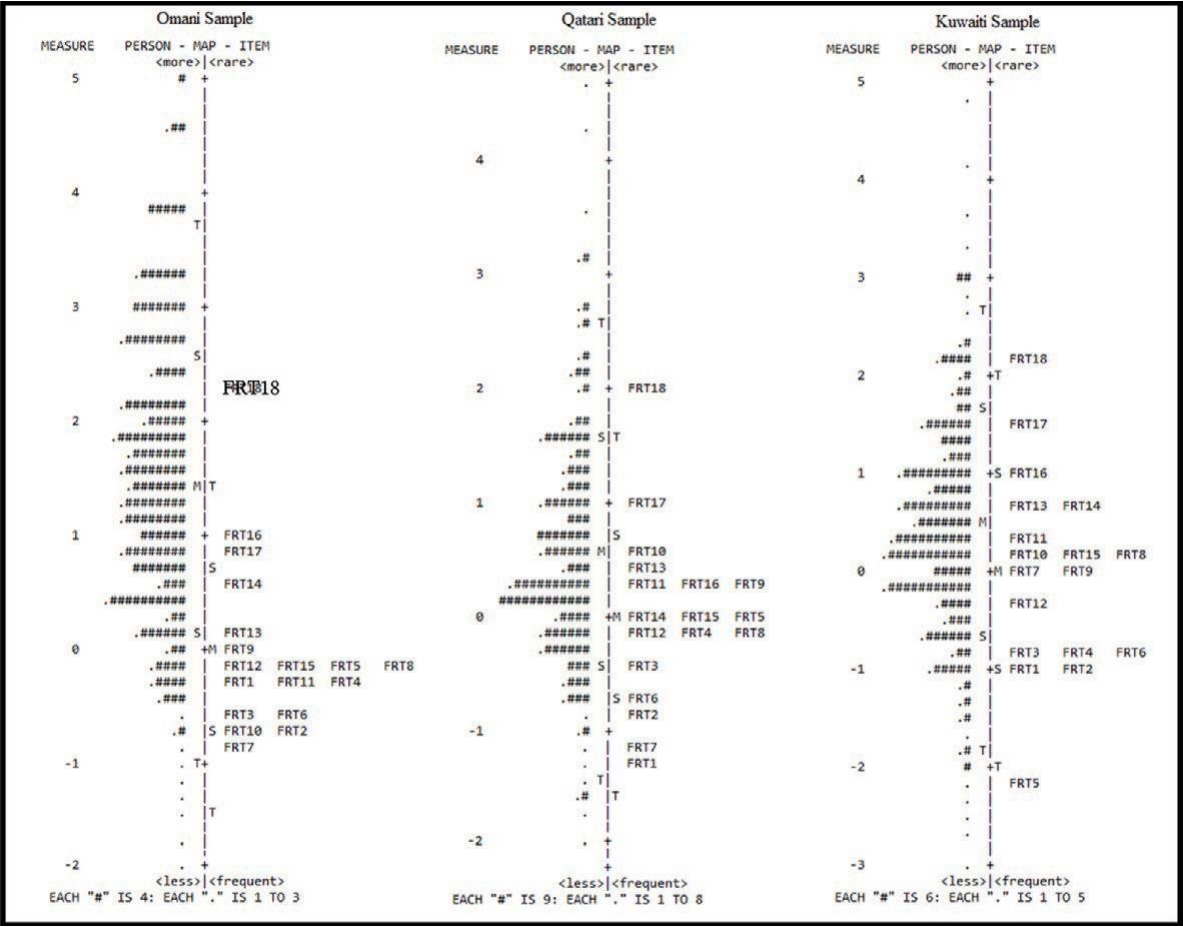
Person–item map for the FRTVMI in the three countries.

A person–item map was constructed to assess the alignment between item difficulty and participant ability levels (see Figure 1). Person–item maps provide a visual representation of how well the distribution of item difficulties corresponds to the distribution of participant abilities along the same latent continuum. The map demonstrated that in the Omani sample, the mean person ability (1.42 logits) exceeded the mean item difficulty, indicating that the items were generally easier for this group. In contrast, the Qatari (0.56 logits) and Kuwaiti (0.45 logits) samples showed a closer alignment between ability and item difficulty, suggesting better targeting [23]. These findings indicate that while the test items adequately covered the ability range of participants in Qatar and Kuwait, the scale appeared somewhat easier for the Omani sample.

### 3.6. Raw Scores and Rasch Ability Estimates

The conversion of raw FRTVMI scores to Rasch ability estimates is presented in Table 5. This conversion allows raw ordinal scores to be expressed on a logit scale, enabling interpretation of performance on an interval‐level metric. By using this table, practitioners can derive Rasch‐based ability estimates corresponding to observed total scores.

**Table 5:** Conversion table from raw sum scores of the FRTVMI to Rasch latent scores.

*Raw score*	Oman			Qatar			Kuwait		
	*θ*	*S.E.*	*Info.*	*θ*	*S.E.*	*Info.*	*θ*	*S.E.*	*Info.*
0	−5.10	1.82	0.30	−5.47	1.83	0.30	−6.44	1.85	0.29
1	−3.92	0.99	1.03	−4.24	1.02	0.97	−5.17	1.05	0.91
2	−3.25	0.69	2.08	−3.52	0.73	1.88	−4.38	0.77	1.69
3	−2.86	0.57	3.12	−3.08	0.61	2.73	−3.89	0.65	2.38
4	−2.59	0.49	4.12	−2.76	0.53	3.54	−3.52	0.58	3.00
5	−2.37	0.44	5.07	−2.50	0.48	4.29	−3.21	0.53	3.56
6	−2.19	0.41	5.96	−2.29	0.45	5.01	−2.95	0.50	4.06
7	−2.03	0.38	6.80	−2.10	0.42	5.69	−2.71	0.47	4.53
8	−1.89	0.36	7.57	−1.93	0.40	6.32	−2.50	0.45	4.95
9	−1.76	0.35	8.29	−1.78	0.38	6.92	−2.31	0.43	5.34
10	−1.65	0.33	8.93	−1.64	0.37	7.49	−2.13	0.42	5.70
11	−1.54	0.32	9.52	−1.52	0.35	8.02	−1.96	0.41	6.04
12	−1.44	0.32	10.04	−1.39	0.34	8.51	−1.80	0.40	6.36
13	−1.34	0.31	10.51	−1.28	0.33	8.97	−1.64	0.39	6.65
14	−1.25	0.30	10.91	−1.17	0.33	9.40	−1.50	0.38	6.93
15	−1.16	0.30	11.26	−1.07	0.32	9.79	−1.35	0.37	7.18
16	−1.07	0.29	11.55	−0.97	0.31	10.15	−1.22	0.37	7.43
17	−0.98	0.29	11.79	−0.87	0.31	10.48	−1.08	0.36	7.66
18	−0.90	0.29	11.98	−0.78	0.30	10.78	−0.96	0.36	7.88
19	−0.82	0.29	12.12	−0.68	0.30	11.05	−0.83	0.35	8.08
20	−0.73	0.29	12.21	−0.59	0.30	11.28	−0.71	0.35	8.27
21	−0.65	0.29	12.26	−0.51	0.30	11.49	−0.59	0.34	8.45
22	−0.57	0.29	12.27	−0.42	0.29	11.66	−0.47	0.34	8.61
23	−0.49	0.29	12.24	−0.34	0.29	11.81	−0.36	0.34	8.75
24	−0.41	0.29	12.17	−0.25	0.29	11.92	−0.24	0.34	8.89
25	−0.33	0.29	12.08	−0.17	0.29	12.00	−0.13	0.33	9.00
26	−0.24	0.29	11.95	−0.08	0.29	12.05	−0.02	0.33	9.09
27	−0.16	0.29	11.79	0.00	0.29	12.07	0.09	0.33	9.17
28	−0.07	0.29	11.61	0.08	0.29	12.06	0.20	0.33	9.23
29	0.01	0.30	11.40	0.16	0.29	12.02	0.31	0.33	9.26
30	0.10	0.30	11.17	0.25	0.29	11.94	0.41	0.33	9.28
31	0.19	0.30	10.93	0.33	0.29	11.83	0.52	0.33	9.27
32	0.29	0.31	10.67	0.42	0.29	11.70	0.63	0.33	9.24
33	0.38	0.31	10.39	0.50	0.29	11.52	0.74	0.33	9.18
34	0.48	0.31	10.10	0.59	0.30	11.32	0.85	0.33	9.09
35	0.58	0.32	9.80	0.68	0.30	11.08	0.96	0.33	8.98
36	0.68	0.32	9.48	0.77	0.30	10.81	1.07	0.34	8.84
37	0.79	0.33	9.16	0.87	0.31	10.50	1.18	0.34	8.67
38	0.90	0.34	8.82	0.96	0.31	10.17	1.30	0.34	8.47
39	1.02	0.34	8.47	1.06	0.32	9.79	1.42	0.35	8.24
40	1.14	0.35	8.11	1.17	0.33	9.39	1.54	0.35	7.98
41	1.26	0.36	7.73	1.28	0.33	8.95	1.67	0.36	7.68
42	1.40	0.37	7.34	1.39	0.34	8.48	1.80	0.37	7.35
43	1.54	0.38	6.93	1.51	0.35	7.97	1.94	0.38	6.98
44	1.69	0.39	6.49	1.64	0.37	7.44	2.09	0.39	6.58
45	1.85	0.41	6.03	1.78	0.38	6.87	2.25	0.40	6.14
46	2.02	0.43	5.53	1.93	0.40	6.26	2.42	0.42	5.66
47	2.21	0.45	4.99	2.10	0.42	5.62	2.60	0.44	5.14
48	2.42	0.48	4.42	2.29	0.45	4.95	2.81	0.47	4.58
49	2.67	0.51	3.81	2.51	0.49	4.24	3.04	0.50	3.97
50	2.95	0.56	3.15	2.77	0.53	3.50	3.32	0.55	3.32
51	3.31	0.64	2.45	3.09	0.61	2.71	3.66	0.62	2.60
52	3.80	0.76	1.71	3.53	0.73	1.87	4.11	0.74	1.81
53	4.58	1.05	0.91	4.26	1.02	0.97	4.86	1.02	0.95
54	5.86	1.86	0.29	5.49	1.84	0.30	6.10	1.84	0.30

*Note:* “*θ*” refers to Rasch ability score; “S.E.” refers to the standard error of estimation; and “Info” refers to the magnitude of information.

**Figure 2:**
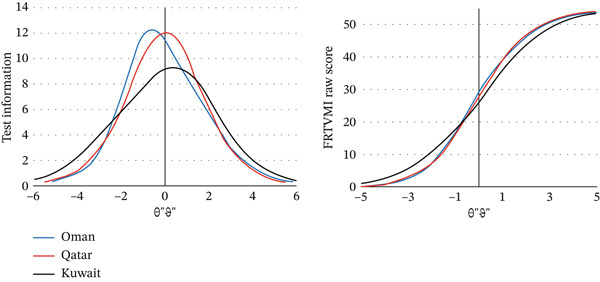
Test information and test characteristic curves for the FRTVMI scale in the three countries.

In addition, test information functions (TIFs) were plotted to examine measurement precision across the ability continuum (see Figure 2). The curves indicate that the FRTVMI provides the greatest measurement information for individuals with VMI abilities between −3 and +3 logits. The test yielded greater information in the Omani and Qatari samples compared with the Kuwaiti sample, suggesting slightly higher measurement precision in those samples [32, 33].

### 3.7. Gender‐Based DIF Analysis

Gender DIF was analyzed using both the M–H chi‐square and the Rasch–Welch *t*‐test, with item classifications following ETS guidelines: Category A (negligible), Category B (slight to moderate), and Category C (moderate to large). Items were considered to exhibit DIF when statistically significant differences were observed between the male and female groups. As shown in Table 6, DIF was detected in 4 items in Oman, 5 in Qatar, and 10 in Kuwait. The Kuwaiti sample displayed the highest number and magnitude of DIF effects, indicating greater gender‐related variability in item functioning within this context.

**Table 6:** DIF analysis by gender for FRTVMI items across the three countries.

	Oman			Qatar			Kuwait		
	Welch‐*t*	M–H chi. sq.	DIF	Welch‐*t*	M–H chi. sq.	DIF	Welch‐*t*	M–H chi. sq.	DIF
FRT1	0.92	1.49	—	1.95	2.67	—	1.82	2.43	—
FRT2	−0.80	0.16	—	0.41	0.01	—	6.78 ^∗∗∗^	41.79 ^∗∗∗^	C*_F_*
FRT3	−1.85	2.37	—	−4.81 ^∗∗∗^	15.40 ^∗∗∗^	C*_M_*	−4.38 ^∗∗∗^	13.61 ^∗∗∗^	C*_M_*
FRT4	−0.45	0.00	—	0.00	0.85	—	−4.18 ^∗∗∗^	17.16 ^∗∗∗^	C*_M_*
FRT5	−0.45	0.36	—	−1.85	0.51	—	−2.35 ^∗^	1.31	A*_M_*
FRT6	0.42	0.03	—	−0.28	0.12	—	3.61 ^∗∗∗^	8.40 ^∗∗^	C*_F_*
FRT7	1.03	0.35	—	1.69	2.87	—	−0.86	0.02	—
FRT8	0.26	0.02	—	−0.96	0.67	—	3.98 ^∗∗∗^	12.08 ^∗∗^	C*_F_*
FRT9	0.00	0.02	—	−2.79 ^∗∗^	4.90 ^∗^	A*_M_*	−5.60 ^∗∗∗^	28.27 ^∗∗∗^	C*_M_*
FRT10	2.88 ^∗∗^	6.76 ^∗∗^	C*_F_*	−0.45	0.02	—	−8.50 ^∗∗∗^	52.99 ^∗∗∗^	C*_M_*
FRT11	1.96	2.65	—	−0.55	1.91	—	1.54	0.85	—
FRT12	−1.14	1.29	—	−2.03 ^∗^	4.51 ^∗^	A*_M_*	−0.80	0.30	—
FRT13	−1.20	1.51	—	1.48	1.54	—	3.94 ^∗∗∗^	15.76 ^∗∗∗^	C*_F_*
FRT14	−1.75	2.98	—	0.00	0.17	—	6.75 ^∗∗∗^	44.65 ^∗∗∗^	C*_F_*
FRT15	2.64 ^∗∗^	4.35 ^∗^	B*_F_*	0.14	0.07	—	−1.53	2.30	—
FRT16	−5.23 ^∗∗∗^	21.95 ^∗∗∗^	C*_M_*	2.48 ^∗^	7.52 ^∗∗^	B*_F_*	1.82	3.35	—
FRT17	3.94 ^∗∗∗^	16.32 ^∗∗∗^	C*_F_*	5.19 ^∗∗∗^	22.71 ^∗∗∗^	C*_F_*	−1.61	2.53	—
FRT18	0.36	0.36	—	1.50	0.02	—	0.00	0.01	—

*Note:* “*A_M_*” refers to negligible DIF favors male; “*B_F_*” refers to slight to moderate DIF favors female; and “*C_F_, C_M_*” refers to moderate to large DIF favors female and male, respectively.

^∗^ *p* < 0.05.

^∗∗^ *p* < 0.01.

^∗∗∗^ *p* < 0.001.

**Figure 3:**
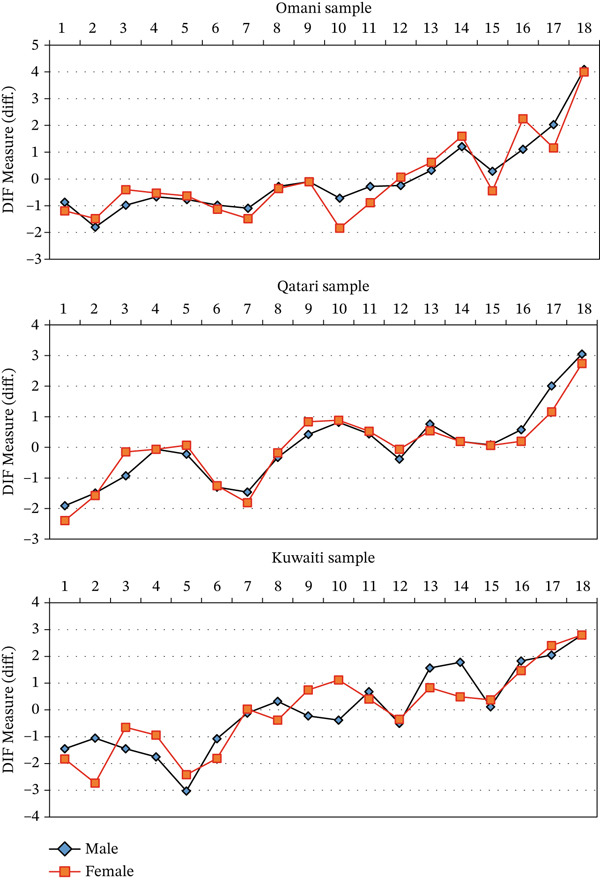
DIF measures by gender for the FRTVMI items in the three countries.

Gender‐based patterns also varied notably across countries (see Figure 3). The identification and classification of DIF were based on the M–H and Rasch–Welch statistical tests reported in Table 6, whereas Figure 3 provides only a graphical illustration of the relative DIF measures for males and females across items. In Oman, Items FRT10, FRT15, and FRT17 favored females, whereas FRT16 favored males. In Qatar, Items FRT16 and FRT17 favored females, and FRT3 favored males. The Kuwaiti sample exhibited the highest number and magnitude of DIF, with five items (FRT2, FRT6, FRT8, FRT13, and FRT14) favoring females, and four items (FRT3, FRT4, FRT9, and FRT10) favoring males. Overall, these results indicate that the magnitude and direction of gender‐related DIF varied across the three national samples.

## 4. Discussion

The Rasch analysis conducted on a sample of adolescents aged 11 years and 1 month to 14 years and 6 months across Oman, Qatar, and Kuwait provides evidence that the items of the FRTVMI measure a coherent underlying construct. Specifically, the Rasch results supported the assumption that the scale operates as a largely unidimensional measure of VMI across the three national samples. The unidimensionality of the scale was supported through multiple Rasch indicators, including fit statistics and PCASR, consistent with recommendations by Brentani and Golia (2007). Establishing unidimensionality is a critical requirement in Rasch measurement because it indicates that item responses are primarily driven by a single latent trait rather than multiple unrelated factors. These results are aligned with prior findings by Emam et al. [9], who reported similar unidimensionality among younger children (6–10 years) across several Arab countries.

To date, no studies have investigated the factorial structure of the FRTVMI in the 11–74 age range, and the official test manual [17] lacks any detailed discussion on its dimensionality, focusing instead on content and criterion‐related validity. This absence of structural validation highlights the contribution of the present Rasch analysis in providing empirical evidence regarding the internal measurement structure of the FRTVMI in adolescent populations. Findings also demonstrated high internal consistency across all three national samples, with item reliability consistently at 0.99 and person reliability ranging from 0.85 to 0.88. These reliability results indicate that the scale produces stable estimates of both item difficulty and participant ability across the three national contexts. These values are consistent with those reported in the test manual (Cronbach′s *α* = 0.87–0.92 for ages 11–15) and comparable with prior international studies, including those by Brown et al. [21] in Australia and Emam et al. [9] in Gulf countries. Furthermore, the person and item separation indices indicate that the FRTVMI effectively distinguishes between different ability levels and item difficulties, suggesting that the scale possesses adequate measurement precision for assessing VMI among adolescents.

Importantly, the study revealed inconsistencies in the hierarchical ordering of items across the three countries when compared with both the manual and the American normative sample. For example, although the manual identifies FRT1 as the easiest item, this was only true for Qatar. In contrast, FRT5 was the easiest for both Oman and Kuwait. Nevertheless, FRT18 consistently emerged as the most difficult item across all contexts, confirming its placement in the original test hierarchy. These results suggest that although the upper end of the item difficulty continuum remains stable, variations occur in the ordering of easier and moderately difficult items across cultural contexts. These cross‐cultural differences—especially in the middle difficulty range—suggest that item functioning is influenced by contextual factors such as cultural norms, educational practices, and familiarity with geometric concepts.

Such variability may also reflect students′ readiness or engagement during the initial test items. Similar findings were noted by Ng et al. [34] in Hong Kong, where item order discrepancies were observed early in the administration. Additionally, variations in reading and writing proficiency—known to correlate with VMI performance [35–37]—may contribute to these effects. Taken together, these findings suggest that the hierarchical structure of the FRTVMI items may not operate identically across educational and cultural contexts. These findings call into question the applicability of a fixed item order across diverse populations and suggest the need for localized recalibration of the test′s administration protocol.

DIF analysis further uncovered gender‐based disparities, particularly in the Kuwaiti sample, where 10 items displayed DIF. The presence of DIF indicates that certain items may function differently for males and females even when individuals possess comparable levels of the underlying ability. These findings are inconsistent with the FRTVMI manual′s assertion that the test is free from gender, racial, and ethnic bias. Although no prior studies have specifically examined DIF in the FRTVMI, the observed gender effects are supported by prior research indicating male disadvantages in fine motor tasks such as handwriting ([38]; Ziviani and Wallen 2006) and VMI performance in Arab contexts [35].

Taken together, these findings underscore the importance of re‐evaluating the FRTVMI for fairness and cultural responsiveness. The results suggest that although the scale demonstrates strong psychometric properties, item functioning may vary across demographic and cultural contexts. Revising test administration procedures and considering localized norms could significantly improve the test′s accuracy, equity, and diagnostic utility in diverse populations.

## 5. Limitations

Several limitations should be acknowledged. First, the study focused exclusively on adolescents (11–16 years), whereas the FRTVMI′s normative data span a broader age range (11–74 years). This discrepancy limits the generalizability of findings to older age groups. Second, although Rasch modeling enabled precise item calibration, the original normative sample did not use Rasch‐based methods, which may partly explain the differences in item hierarchy.

Third, the study sample comprised typically developing students from general education settings in three Gulf countries, excluding individuals with developmental or neurological conditions. As such, the diagnostic applicability of the FRTVMI in clinical contexts remains to be determined. Future studies should include clinical populations to enhance the generalizability of findings.

Finally, the results call for the development of localized Rasch‐calibrated norms for the Gulf region to ensure the cultural validity and fairness of the FRTVMI. Such efforts would improve the interpretive accuracy of test scores and facilitate more equitable assessment of visual–motor skills in diverse educational contexts.

## 6. Conclusion

This study provides an empirical evidence supporting the psychometric validity of the FRTVMI for assessing VMI among adolescents in Oman, Qatar, and Kuwait. Rasch analysis indicated that the scale functions as a largely unidimensional measure with strong item reliability and satisfactory person reliability across the three national samples. The results also revealed cross‐cultural differences in the hierarchical ordering of several items and identified gender‐related DIF, particularly in the Kuwaiti sample. These findings suggest that although the FRTVMI demonstrates strong measurement properties, certain items may operate differently across cultural and demographic contexts. Accordingly, future research should consider the development of localized Rasch‐calibrated norms and further evaluation of item functioning to enhance the fairness and interpretive accuracy of the test in diverse populations.

## Funding

This study was supported by Sultan Qaboos University (10.13039/501100004351) (IG/EDU/PSYC/22/03).

## Conflicts of Interest

The authors declare no conflicts of interest.

## Data Availability

The datasets analyzed during the current study are available from the corresponding author on reasonable request.
